# Hepatocyte-specific contrast agent-enhanced magnetic resonance cholangiography: perioperative evaluation of the biliary tree

**DOI:** 10.1590/0100-3984.2015.0213

**Published:** 2017

**Authors:** Luciana Carmen Zattar-Ramos, Regis Otaviano Franca Bezerra, Luis Tenório de Brito Siqueira, Marcos Roberto Menezes, Claudia da Costa Leite, Giovanni Guido Cerri

**Affiliations:** 1 MD, Radiologist, Hospital Sírio-Libanês, São Paulo, SP, Brazil.; 2 MD, Radiologist, Hospital Sírio-Libanês and Instituto do Câncer do Estado de São Paulo (Icesp), São Paulo, SP, Brazil.; 3 MD, Radiologist, Hospital Regional de Presidente Prudente and Hospital Nossa Senhora das Graças, Presidente Prudente, SP, Brazil.; 4 PhD, MD, Radiologist, Hospital Sírio-Libanês and Instituto do Câncer do Estado de São Paulo (Icesp), São Paulo, SP, Brazil.; 5 Associate Professor in the Department of Radiology and Oncology of the Faculdade de Medicina da Universidade de São Paulo (FMUSP), Radiologist, Hospital Sírio-Libanês, São Paulo, SP, Brazil.; 6 Full Professor in the Department of Radiology and Oncology of the Faculdade de Medicina da Universidade de São Paulo (FMUSP), Radiologist, Hospital Sírio-Libanês, São Paulo, SP, Brazil.

**Keywords:** Biliary tree, Magnetic resonance imaging, Hepatobiliary-specific contrast agents, Vias biliares, Ressonância magnética, Contrastes hepatoespecíficos

## Abstract

A large number of gadolinium chelates have recently been tested in clinical trials. Some of those have already been approved for clinical use in the United States and Europe. Thus, new diagnostic perspectives have been incorporated into magnetic resonance imaging studies. Among such gadolinium chelates are hepatobiliary-specific contrast agents (HSCAs), which, due to their property of being selectively taken up by hepatocytes and excreted by the biliary ducts, have been widely used for the detection and characterization of focal hepatic lesions. In comparison with conventional magnetic resonance cholangiography (MRC), HSCA-enhanced MRC provides additional information, with higher spatial resolution and better anatomic evaluation of a non-dilated biliary tree. A thorough anatomic assessment of the biliary tree is crucial in various hepatic surgical procedures, such as complex resection in patients with colorectal cancer and living-donor liver transplantation. However, the use of HSCA-enhanced MRC is still limited, because of a lack of data in the literature and the poor familiarity of radiologists regarding its main indications. This pictorial essay aims to demonstrate the use of HSCA-enhanced MRC, with particular emphasis on anatomical analysis of the biliary tree, clinical applications, and the most important imaging findings.

## Abstract (pt)

Recentemente, um grande número de quelantes de gadolínio tem sido testado em ensaios laboratoriais. Alguns deles já foram inclusive aprovados para uso clínico nos Estados Unidos e na Europa. Com isso, novas perspectivas diagnósticas foram incorporadas nos estudos de ressonância magnética. Dentre estes quelantes de gadolínio, os contrastes hepatobiliares específicos (CHBEs) têm sido amplamente utilizados para a caracterização e detecção de lesões focais hepáticas, essencialmente pela propriedade de serem captados pelos hepatócitos e excretados na via biliar. Além disso, os CHBEs trouxeram novas informações na avaliação da árvore biliar quando comparados à colangiorressonância convencional, proporcionando uma maior resolução espacial e melhor avaliação da anatomia da árvore biliar não dilatada. A avaliação da anatomia biliar é de fundamental importância em cirurgias hepáticas, como ressecções complexas em tumores colorretais ou no transplante hepático com doador vivo, porém, o uso dos CHBEs ainda é restrito para estes propósitos. Em razão da escassa literatura sobre o tema e da pouca familiaridade dos radiologistas com as principais indicações, o presente ensaio iconográfico tem por objetivo demonstrar o uso de CHBEs na avaliação perioperatória das vias biliares, ressaltando a avaliação anatômica, as indicações e os principais achados de imagem.

## INTRODUCTION

Gadolinium-based hepatocyte-specific contrast agents (HSCAs) allow the functional and anatomical assessment of the biliary tract, thus facilitating the planning of hepatobiliary surgery and the diagnosis of any associated postoperative complications^(1-6)^. Hepatobiliary surgery involves procedures of great technical difficulty. In many cases, complications related to the biliary tract occur and can be accompanied by unexpected anatomical variations. In liver transplantation, the rate of such complications ranges from 10% to 25%, fatal complications occurring in up to 10% of cases^(7)^.

Conventional T2-weighted magnetic resonance cholangiography (MRC) has the disadvantage of assessing only the anatomy of the biliary tract, providing little information in cases in which there is no dilatation. Therefore, some studies suggest that a thorough evaluation of the biliary system involves a combination of anatomical and functional evaluation techniques^(8)^.

The HSCAs approved for clinical use in Brazil^(9)^, unlike the contrast agents routinely used in magnetic resonance imaging, are selectively captured by functioning hepatocytes and have high rates of elimination (of approximately 50%) through the biliary tract^(1-5,9)^. The MRC examination protocols should be adapted for time optimization, because it is necessary to obtain late images at 20-30 min after injection of the HSCA^(5,6,10-12)^.

The aim of the present study was to demonstrate the use of HSCA-enhanced MRC in the perioperative evaluation of the bile ducts. We highlight the main imaging aspects that can inform the practice.

## METHODS

We selected MRC examinations in which the HSCA gadoxetic acid (Primovist^®^) was used for the perioperative assessment of the upper abdomen in patients undergoing hepatobiliary surgery between January 2013 and February 2015. The images were obtained from the digital archives of our institution.

## EVALUATION OF THE BILIARY ANATOMY

**Figure 1:**
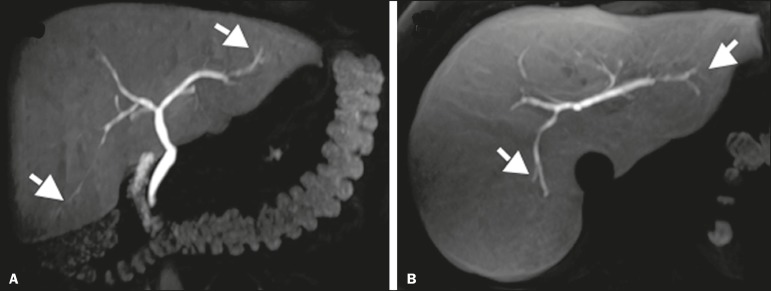
Preoperative evaluation of a liver donor candidate. The use of HSCA-enhanced MRC allows an excellent evaluation of the normal anatomy of the biliary tree, with delineation of the subsegmental ducts (arrows).

Anatomical variations of the biliary tract occur in approximately 30% of individuals. The correct characterization of such variations is necessary in order to avoid iatrogenic injuries during hepatobiliary surgery and procedures, especially in cases of living donors for liver transplantation (Figure 1), in which accurate knowledge of the biliary anatomy is essential^(2,4-6,13)^.

**Figure 2:**
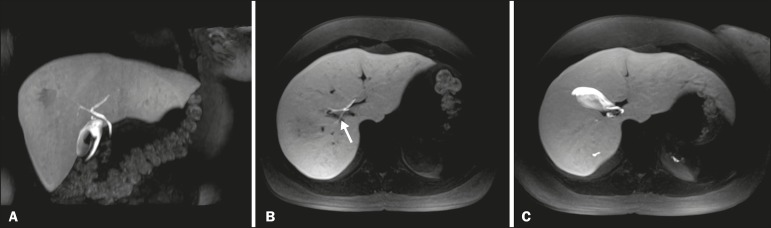
Preoperative evaluation for right hepatectomy in a patient with metastatic colorectal cancer. HSCA-enhanced MRC provides excellent delineation of the biliary tree, demonstrating an accessory bile duct in segment VII (arrow), the recognition of which is important for the surgical planning.

Delineation of the biliary tree occurs in the late hepatobiliary phase of acquisition, at 20-30 min after HSCA administration. The MRC sequences acquired with HSCA are T1-weighted, providing images with better spatial resolution than those obtained in the T2-weighted sequences employed in conventional (non-contrast-enhanced MRC, especially when there is no dilatation of the bile ducts^(5,6,10-12)^, as illustrated in Figure 2.

## DIFFERENTIATION BETWEEN BILIARY AND NONBILIARY LESIONS

**Figure 3:**
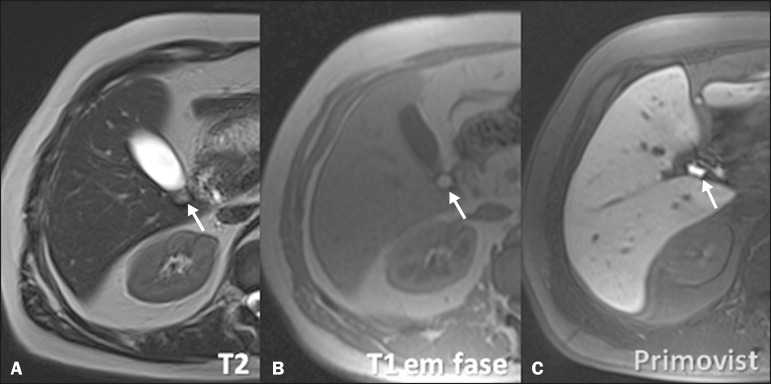
MRC showing a small cystic formation (arrows), adjacent to the common hepatic duct, in a T2-weighted sequence (A) and a non-contrast-enhanced T1-weighted in-phase sequence (B). An HSCA-enhanced T1-weighted MRC sequence (C) unequivocally demonstrates the diagnosis of bile cyst, after its filling with contrast (arrow).

Making a distinction between cystic lesions near the biliary tree and those actually within the biliary tract is important in clinical management. The presence of the HSCA within the lumen of a cystic lesion confirms its biliary origin (Figure 3) and allows the differentiation among choledochal cysts, pericholedochal cystic formations, diverticula, and duodenal duplications^(4,5,11)^.

## DIAGNOSIS OF ACUTE CHOLECYSTITIS

**Figure 4:**
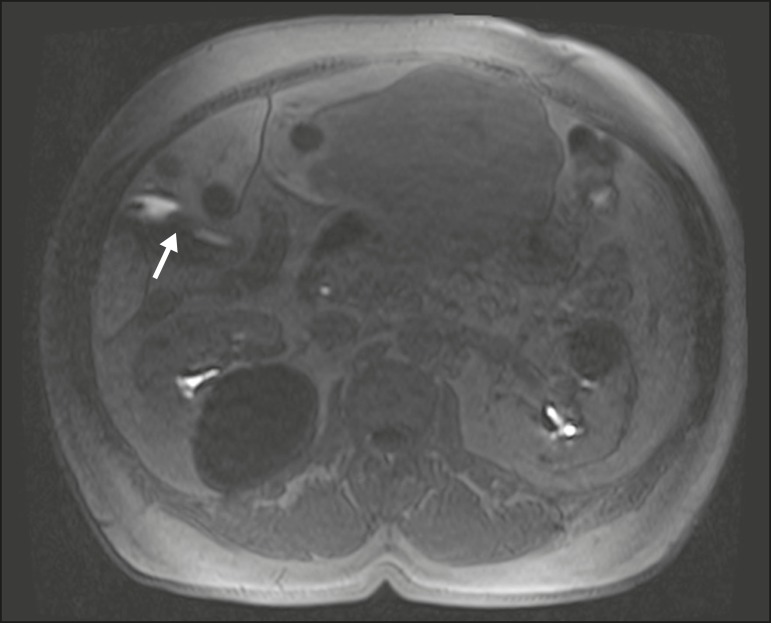
Case of choledocholithiasis. An HSCA-enhanced MRC scan shows a lack of contrast uptake within the gallbladder (arrow).

**Figure 5:**
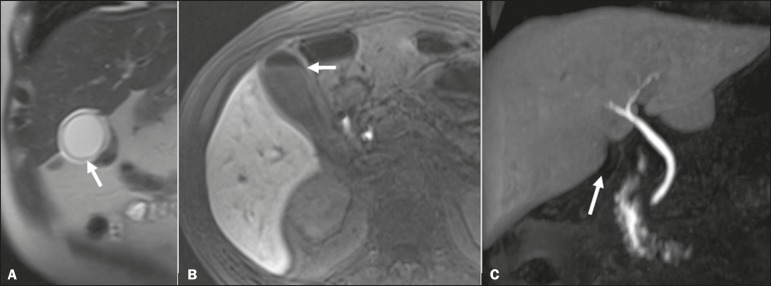
Acute cholecystitis. Fast spin-echo axial T2-weighted MRC sequence (A) demonstrating parietal thickening (arrow); non-contrast-enhanced axial T1-weighted MRC sequence (B) showing high-protein biliary content forming a level with a small amount of gas (arrow); and HSCA-enhanced coronal T1-weighted MRC sequence (C) showing no contrast uptake within the gallbladder (arrow). In such cases, the use of morphine can be considered, although it is rarely necessary, given the set of findings suggestive of cholecystitis.

Acute cholecystitis is a common cause of acute abdomen and is initially investigated by ultrasound. However, when the ultrasound findings are inconclusive, HSCA-enhanced MRC can be useful, replacing scintigraphy with diisopropyl iminodiacetic acid (DISIDA scintigraphy). A lack of HSCA uptake in the gallbladder during MRC constitutes a specific sign of acute cholecystitis (similar to that seen in DISIDA scintigraphy), with the additional advantage of allowing evaluation of the differential diagnoses of abdominal pain^(4-6,11,13,14)^. It is noteworthy that the functional evaluation is considered in combination with the other usual MRC aspects, such as choledocholithiasis (Figure 4), perfusion disorder (Figure 5), gallbladder thickening, and peribiliary fluid collections^(2-4,6,8)^.

## DIAGNOSIS AND GRADING OF BILIARY OBSTRUCTIONS

**Figure 6:**
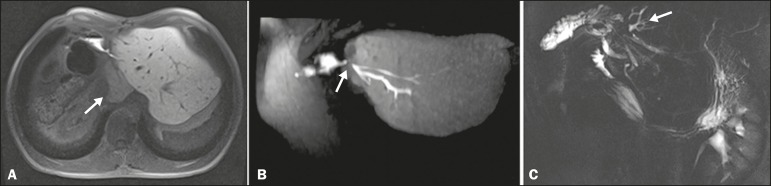
Postoperative evaluation of a hepatectomy patient with biliodigestive anastomosis. An HSCA-enhanced MRC scan shows late contrast uptake in the caudate lobe (A, arrow), suggesting severe obstruction (B, arrow), even before there was significant dilation of the biliary tree. An MRC scan acquired three months later showed mild ectasia of the intrahepatic bile ducts of the caudate lobe with no stricture development in the lateral ducts of the left lobe (C, arrow).

**Figure 7:**
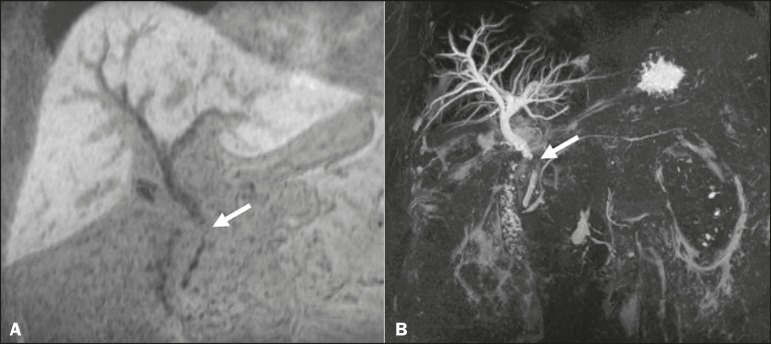
HSCA-enhanced coronal T1-weighted MRC sequence acquired in the late phase (A) showing that, despite moderate dilation of the biliary tract, there was no elimination of contrast in the hepatobiliary phase, due to obstruction of the distal bile duct (arrow). Non-contrast-enhanced coronal MRC scan (B).

Changes in the HSCA dynamics in the biliary tract allow the early diagnosis of obstruction, even without evident dilatation (Figure 6), as well as allowing its classification^(2,4,6,10-14)^, as follows: complete biliary obstruction-absence of contrast uptake in the distal and proximal portions of a stricture or obstructive lesion (Figure 7); nearly complete biliary obstruction-contrast uptake that is significantly late and only in the proximal portion of a stricture or obstructive lesion; or partial biliary obstruction-contrast uptake only outside the area of the stricture or obstructive lesion. Caution should be exercised when evaluating patients with markedly impaired hepatic function, because the dysfunction changes the way in which the HSCA is metabolized, reducing its uptake in the biliary tract and simulating obstructive processes^(6,10,12)^. Early, accurate grading and characterization of biliary obstructions is important in order to guide the therapeutic practice and, occasionally, the surgical planning^(2,4,6,10-14)^.

## POSTOPERATIVE EVALUATION OF THE BILIARY TRACT

Iatrogenic injuries are the most common complications associated with hepatobiliary surgery, especially performed by laparoscopy. The most common iatrogenic injuries are fistulas, which can occur in the immediate or early postoperative period, and strictures, which occur later^(4-6,11)^.

**Figure 8:**
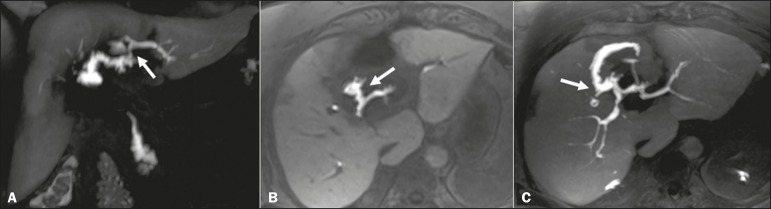
Images acquired in the hepatobiliary phase of HSCA excretion. Note the extravasation of the contrast medium from the main duct of the lateral segment of the left lobe (arrows).

The use of HSCA-enhanced MRC allows the diagnosis of extravasations through their direct visualization, as well as demonstrating their origin (Figure 8). Bile duct injury is graded according to the classification system devised by Bismuth, as well as that subsequently devised by Strasberg^(4-6,11)^.

**Figure 9:**
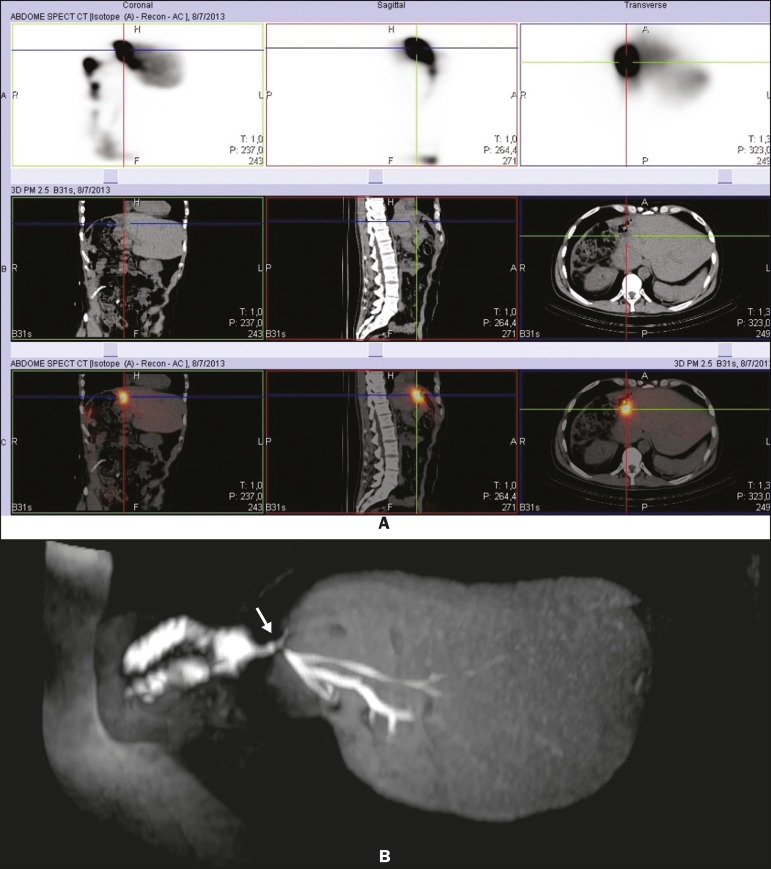
Postoperative evaluation of a hepatectomy patient with biliodigestive anastomosis. DISIDA scintigraphy suggested a liquid collection in the anastomosis plane (A). An HSCA-enhanced MRC scan showed anastomosis integrity and contrast accumulation in the “J” pouch (B, arrow), clearly disproving the hypothesis of a collection or fistula.

The combination of conventional (T2-weighted) MRC and HSCA-enhanced MRC has a reported accuracy of 84% for the detection of biliary fistulas^(8)^. Therefore, the combined use of those two techniques increases the likelihood of the correct identification and localization of the extravasation site (Figure 9)^(4-6,11)^.

## DIFFERENTIAL DIAGNOSIS BETWEEN ABDOMINAL COLLECTIONS AND BILIARY FISTULAS (BILOMAS)

**Figure 10:**
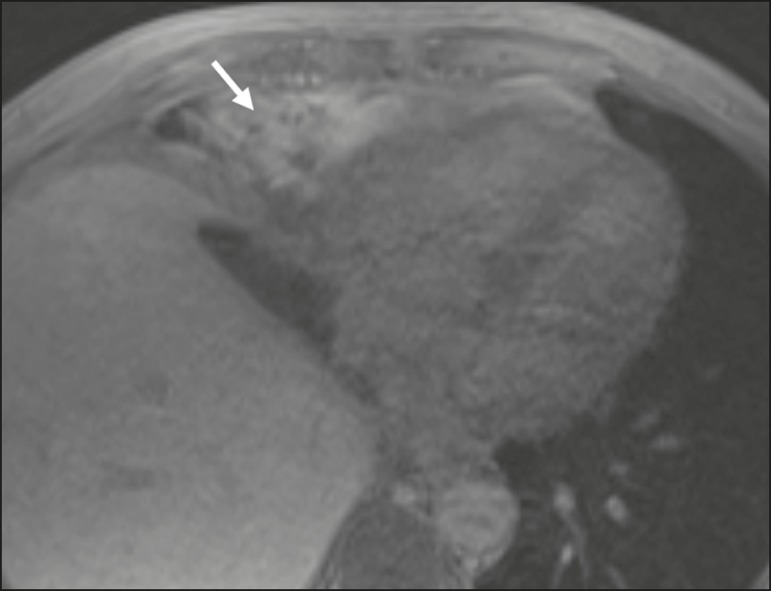
Collection adjacent to the hepatectomy site, which fills with contrast medium in the hepatobiliary phase of HSCA excretion, confirming the diagnosis of biloma (arrow).

Bilomas are collections of bile outside of the biliary tree. They can be spontaneous, traumatic, or iatrogenic, the majority (70%) occurring in the upper right quadrant of the abdomen. The accurate characterization of a biloma allows it to be distinguished from collections of other natures, as well as informing decisions regarding its management. A late image after HSCA administration confirms that the collection is composed of bile and allows the site of extravasation to be identified^(4,5,11)^. The use of MRC with a contrast agent that is specific for biliary content allows better differentiation among abdominal collections, loculated ascites, and cystic formations adjacent to the bile ducts (Figure 10), providing important additional information in relation to conventional T2-weighted MRC^(4,5,11)^.

## CONCLUSION

The use of HSCA-enhanced MRC is an important tool that adds functional information in the perioperative assessment of the biliary tree and is complementary to T2-weighted MRC^(3-6)^. The correct indication and interpretation of the images can alter the clinical practice, as well as precluding the need for other diagnostic tests, invasive procedures, biopsies, or additional interventions^(2,11,12)^.

## References

[1] Guglielmo FF, Mitchell DG, Gupta S (2014). Gadolinium contrast agent selection and optimal use for body MR
imaging. Radiol Clin North Am.

[2] Van Beers BE, Pastor CM, Hussain HK (2012). Primovist, Eovist what to expect?. J Hepatol.

[3] Ringe KI, Husarik DB, Gupta RT (2011). Hepatobiliary transit times of gadoxetate disodium (Primovist(r))
for protocol optimization of comprehensive MR imaging of the biliary
system-what is normal. Eur J Radiol.

[4] Gupta RT (2013). Evaluation of the biliary tree and gallbladder with
hepatocellular MR contrast agents. Curr Probl Diagn Radiol.

[5] Gupta RT, Brady CM, Lotz J (2010). Dynamic MR imaging of the biliary system using
hepatocyte-specific contrast agents. AJR Am J Roentgenol.

[6] Seale MK, Catalano O, Saini S (2009). Hepatobiliary-specific MR contrast agents role in imaging the
liver and biliary tree. Radiographics.

[7] Hyodo T, Kumano S, Kushihata F (2012). CT and MR cholangiography advantages and pitfalls in
perioperative evaluation of biliary tree. Br J Radiol.

[8] Kantarci M, Pirimoglu B, Karabulut N (2013). Non-invasive detection of biliary leaks using
Gd-EOB-DTPA-enhanced MR cholangiography comparison with T2-weighted MR
cholangiography. Eur Radiol.

[9] Bittencourt LK, Hausmann D, Gasparetto EL (2012). Ressonância magnética do fígado com
contraste hepato-específico experiência clínica inicial
no Brasil. Rev Col Bras Cir.

[10] Goodwin MD, Dobson JE, Sirlin CB (2011). Diagnostic challenges and pitfalls in MR imaging with
hepatocyte-specific contrast agents. Radiographics.

[11] Lee NK, Kim S, Lee JW (2009). Biliary MR imaging with Gd-EOB-DTPA and its clinical
applications. Radiographics.

[12] Reimer P, Schneider G, Schima W (2004). Hepatobiliary contrast agents for contrast-enhanced MRI of the
liver properties, clinical development and applications. Eur Radiol.

[13] Lee SW, Cha SH, Chung HH (2014). Functional magnetic resonance cholangiography with Gd-EOB-DTPA a
study in healthy volunteers. Magn Reson Imaging.

[14] Flancbaum L, Choban PS, Sinha R (1994). Morphine cholescintigraphy in the evaluation of hospitalized
patients with suspected acute cholecystitis. Ann Surg.

